# *FGFR1 *is amplified during the progression of *in situ *to invasive breast carcinoma

**DOI:** 10.1186/bcr3239

**Published:** 2012-08-03

**Authors:** Min Hye Jang, Eun Joo Kim, Yoomi Choi, Hee Eun Lee, Yu Jung Kim, Jee Hyun Kim, Eunyoung Kang, Sung-Won Kim, In Ah Kim, So Yeon Park

**Affiliations:** 1 Department of Pathology, Seoul National University College of Medicine, 103 Daehakro, Jongno-gu, Seoul 110-799, Korea; 2 Department of Pathology, Seoul National University Hospital, 101 Daehakro, Jongno-gu, Seoul 110-744, Korea; 3 Department of Pathology, Seoul National University Bundang Hospital, 300 Gumi-dong, Bundang-gu, Seongnam, Gyeonggi 463-707, Korea; 4 Breast Care Center, Seoul National University Bundang Hospital, 300 Gumi-dong, Bundang-gu, Seongnam, Gyeonggi 463-707, Korea

## Abstract

**Introduction:**

Gene amplification is an important mechanism for activating oncogenes in malignant tumors. Although amplification of *HER2*, *C-MYC*, *CCND1 *and *FGFR1 *has been reported in breast cancers, their role in the progression of *in situ *to invasive breast carcinoma is unclear. To investigate this question we compared the amplification frequencies of these genes in pure ductal carcinoma *in situ *(DCIS), DCIS associated with invasive carcinoma, and invasive carcinoma.

**Methods:**

We performed fluorescence *in situ *hybridization of the selected genes on tissue microarrays composed of 179 pure DCIS and 438 invasive carcinomas. Two hundred and sixteen of the latter had DCIS components, and in those cases we compared gene amplification in the intraductal and invasive components of each carcinoma.

**Results:**

The rate of amplification of *FGFR1 *was higher in invasive carcinomas than in the pure DCIS, but the opposite was true for *HER2 *amplification. These findings applied consistently to high-grade tumors, but not to low/intermediate-grade tumors. The amplification status of *HER2*, *C-MYC*, *CCND1 *and *FGFR1 *was generally similar in the matched invasive and DCIS components of the same tumors. However, *FGFR1 *amplification was more common in the invasive components than in the DCIS components. In survival analyses, *FGFR1 *amplification was found to be an independent prognostic factor for poor disease-free survival for all patients with invasive carcinoma and for the hormone receptor-positive subgroup.

**Conclusion:**

Amplification of *HER2*, *C-MYC *and *CCND1 *seems to play a role in the early development of breast cancer, but not in its progression. However, the increased frequency of *FGFR1 *amplification in invasive carcinomas compared with pure DCIS and in the invasive components of individual tumors, and its association with decreased disease-free survival, suggests a role for *FGFR1 *amplification in the progression of breast cancer including *in situ*-to-invasive transition, as well as initiation.

## Introduction

Development of breast cancer depends on the accumulation of a variety of genetic alterations, including activation or amplification of oncogenes [1]. In breast cancer, the most prominent and frequent amplicons have been located at chromosomal positions 1q, 8p12, 8q24, 11q13, 12p13, 16p13, 7q12-21 and 20q13, and several target oncogenes have been identified [2-5]. The best characterized oncogene is *HER2*, located at 17q12-21, which is amplified in 15 to 20% of breast cancers [6,7]. Other oncogenes that are frequently amplified in breast cancer include *C-MYC*, *FGFR1 *and *CCND1*. *C-MYC*, located at 8q24, encodes a transcription factor with a basic region/helix-loop-helix/leucine zipper domain, which is a key regulator of cell growth, proliferation, metabolism, differentiation, and apoptosis [8]. The frequency of *C-MYC *amplification in breast cancer varies, with an average frequency of 16% [9]. Although *C-MYC *amplification is associated with a risk of relapse and death [9-12], its prognostic role is still unclear. *FGFR1 *at 8p12 encodes a tyrosine kinase receptor belonging to the fibroblast growth factor and growth factor receptor family, which is amplified in 9 to 15% of breast cancers [13-16]. *FGFR1 *amplification is associated with a poor prognosis in breast cancer [14,16,17]. *CCND1*, located at 11q13, is an estrogen-responsive gene with oncogenic potential as it influences the G_1_/S phase transition [18]. *CCND1*is amplified in 13 to 20% of breast cancers [2,16,19].

Ductal carcinoma *in situ *(DCIS) is an early pathologic stage of breast cancer characterized by proliferation of tumor cells within the ductal-lobular system but not extending through the basement membrane. Like invasive breast cancer, DCIS comprises a highly heterogeneous group of diseases with diverse histologic features, molecular alterations and risks of progression to invasive cancer [20-22]. There appear to be at least two broad groups of DCIS lesions: low-grade DCIS characterized by 16q loss and 1q gain, and high-grade DCIS displaying complex genetic changes including gains at chromosome locations 8q and 17q [23-25]. Molecular studies have revealed that *in situ *lesions preferentially cluster with invasive lesions of the same grade in gene expression profiling, and that the *in situ *and invasive components of the same tumor exhibit similar patterns of genetic alterations, suggesting that DCIS is a precursor for invasive cancer of similar grade [23,25,26].

The natural history of DCIS is poorly understood, although it is known that 14 to 53% of *in situ *lesions evolve to invasive cancer over a period of 10 years or more if left untreated [20]. The mechanisms by which DCIS progress to invasive carcinomas are not well understood, and robust biomarkers capable of stratifying the aggressive forms of DCIS from the indolent forms are lacking. Moreover, the role of gene amplification in the progression of DCIS to invasive breast cancer is uncertain. Some workers found no difference in gene amplification frequencies between DCIS and invasive carcinomas [27-29]. Others have suggested that *C-MYC *amplification plays an important role in the transition, because they found amplification only in the invasive component [30,31]. However, this finding was not confirmed in other studies [27-29]. Furthermore, there have been few studies comparing amplification of *FGFR1 *in pure DCIS, DCIS associated with invasive cancer, and invasive breast cancer, although it has been found to be associated with breast cancer progression [14,16,17].

In this study, we compared the gene amplification frequencies of *HER2*, *C-MYC*, *CCND1 *and *FGFR1 *in a relatively large series of pure DCIS, DCIS associated with invasive carcinoma, and invasive carcinomas, to investigate the role of gene amplification in the progression of DCIS to invasive carcinomas. We also analyzed the gene amplification status of the *in situ *and invasive components in the invasive carcinomas that were accompanied by DCIS.

## Materials and methods

### Tissue specimens

Six hundred and seventeen consecutively resected primary breast cancers including 438 invasive carcinomas and 179 DCIS were collected in Seoul National University Bundang Hospital from 2003 to 2009. Of the 438 invasive breast cancers, 216 cases had enough DCIS component for evaluation. The baseline characteristics of the cases are summarized in Table 1. Clinicopathologic information was obtained by reviewing medical records and H & E-stained sections. The following histopathologic variables of the invasive carcinomas were determined: histologic subtype, T stage, N stage, Bloom-Richardson histologic grade, lymphovascular invasion, tumor border, and presence or absence of a DCIS component. For DCIS cases, we recorded the extent of tumor, nuclear grade, presence of necrosis and architectural pattern. All cases were independently reviewed by two breast pathologists (SYP and HEL). The study was approved by the institutional review board of Seoul National University Bundang Hospital (Protocol # B-0909/083-002), waiving the requirement for informed consent for the study.

**Table 1:** Baseline characteristics of specimens

Characteristic	*n *(%)
**Pure DCIS**	179
Age (years)	
Mean (range)	50 (26 to 82)
Grade	
Low	16 (8.9)
Intermediate	85 (47.5)
High	78 (43.6)
**Invasive carcinomas**	438
Age (years)	
Mean (range)	50 (21 to 87)
Stage	
I	143 (32.6)
II	235 (53.7)
III	55 (12.6)
IV	5 (1.1)
Histologic subtype	
Invasive ductal carcinoma	397 (90.6)
Invasive lobular carcinoma	14 (3.2)
Mucinous carcinoma	12 (2.7)
Metaplastic carcinoma	5 (1.1)
Tubular carcinoma	3 (0.7)
Others	7 (1.6)
Histologic grade	
Grade I	88 (20.1)
Grade II	148 (33.8)
Grade III	188 (42.9)
Not determined	14 (3.2)
Grade of associated DCIS^a^	
Low	10 (4.6)
Intermediate	100 (46.3)
High	106 (49.1)

^a^From 216 ductal carcinoma *in situ *(DCIS) associated with invasive carcinoma.

### Tissue microarray construction

We used tissue microarrays (TMAs) to assess the gene amplification status in the collected cases. To overcome sampling errors caused by TMA evaluation, all slides including slides immunohistochemically stained for standard biomarkers were reviewed and the most representative tumor section was selected for each case. If the tumor showed regional differences in histology or biomarker expression, different tumor areas were selected. Three tissue columns of invasive carcinoma and pure DCIS (2.0 mm in diameter for invasive carcinomas, 4.0 mm in diameter for DCIS) were taken from different areas of the tumors and arranged in new TMA blocks using a trephine apparatus (Superbiochips Laboratories, Seoul, Korea). Lots of DCIS associated with invasive carcinoma were not large enough for multi-core construction, and one tissue column of DCIS associated with invasive carcinoma (4.0 mm in diameter) was selected. However, because the analysis of DCIS associated with invasive carcinoma in a single TMA core may not be representative, we re-evaluated the discrepant cases for gene amplification between DCIS and invasive components of the same tumor using all tumor sections with a DCIS component.

### Fluorescence *in situ *hybridization assays

To characterize *C-MYC *and *CCND1 *amplification, we performed fluorescence *in situ *hybridization (FISH) analyses on TMA samples with commercially available locus-specific probes and chromosome enumeration probes (CEPs): LSI C-MYC SpectrumOrange probe (8q24.12-q24.13) and CEP 8 SpectrumGreen probe (8p11.1-q11.1); and LSI CCND1 SpectrumOrange probe (11q13) and CEP 11 SpectrumGreen probe (11p11.11-q11)] (Abbott Molecular, Downers Grove, IL, USA). *HER2 *FISH was performed using the PathVysion assay (Abbott Molecular), and *FGFR1 *amplification was analyzed with locus-specific BAC, RP11-100B16 (chr8:38,358,839-38,522,417) and CEP 8 SpectrumGreen probes (8p11.1-q11.1) (Abbott Molecular). The BAC clone was obtained from Invitrogen (Carlsbad, CA, USA) and purified with a large construction kit (Qiagen, Valencia, CA, USA). DNA from the BAC clone was labeled with SpectrumOrange using a nick translation kit (Abbott Molecular), and the specificity of the BAC probe was verified by metaphase FISH analyses to confirm their proper chromosomal localization and rule out the presence of any cross-hybridization.

FISH was performed as reported for analysis of *HER2 *amplification [32]. Briefly, 4 μm deparaffinized TMA sections were incubated in pretreatment solution (Abbott Molecular) at 80°C for 30 minutes, then in protease solution (Abbott Molecular) for 20 minutes at 37°C. Probes were diluted in tDen-Hyb-2 hybridization buffer (InSitus Biotechnologies, Albuquerque, NM, USA). Co-denaturation of the probes and DNA of the tissue sections was achieved by incubating for 5 minutes (73°C for *HER2*, 80°C for *C-MYC *and *CCND1*, 90°C for *FGFR1*) using HYBrite™ (Abbott Molecular) followed by 16-hour hybridization at 37°C. Post-hybridization washes were performed according to the protocols supplied. Slides were mounted in 4',6-diamidino-2-phenylindole/anti-fade and viewed with a fluorescence microscope. Gene signals per cell were evaluated in 50 tumor nuclei for each TMA core. The average gene copy number and gene:CEP ratio was calculated separately for each core, and the tumor was considered amplified if the average gene copy number was > 6.0 or if the gene:CEP signal ratio was > 2.2 in at least a core. In addition to the equivocal cases, 10% of all cases were scored independently by two observers (MHJ and EJK).

### Immunohistochemical analyses and scoring

Expression of standard biomarkers including estrogen receptor (ER), progesterone receptor (PR), HER2, p53 and Ki-67 was evaluated in full sections at the time of diagnosis or in TMA sections for missing data during the study. Epidermal growth factor receptor (EGFR) and cytokeratin 5/6 were evaluated using TMAs. Tissue sections 4 μm thick were cut, dried, deparaffinized, and rehydrated following standard procedures. All of the sections were subjected to heat-induced antigen retrieval. Immunohistochemical staining was carried out in a BenchMark XT autostainer (Ventana Medical Systems, Tucson, AZ, USA) using an i-View detection kit (Ventana Medical Systems) for ER (1:100, clone SP1; Labvision, Fremont, CA, USA), PR (1:70, PgR 636; Dako, Capinteria, CA, USA), HER2 (1:700, polyclonal; Dako), p53 (1:600, D07; Dako), Ki-67 (1:250, MIB-1; Dako), cytokeratin 5/6 (1:50, clone D5/16 B4; Dako) and EGFR (EGFR pharmDx™; Dako).

ER and PR were regarded positive if there were at least 1% positive tumor nuclei, according to the American Society of Clinical Oncology/College of American Pathologists guidelines [33]. Expression of HER2 and EGFR was scored as follows: 0, no staining; 1+, weak and incomplete membranous staining in ≥ 10% of the tumor cells; 2+, weak to moderate, complete membranous staining in ≥ 10% of the tumor cells; and 3+, strong, complete membranous staining in ≥ 30% of the tumor cells. Any positive staining was regarded as positive for EGFR, and 3+ was considered positive for HER2. For cytokeratin 5/6, cases with any positive membranous staining were grouped as positive. For p53, cases with 10% or more positive staining were grouped as positive. For the Ki-67 proliferation index, cases with 20% or more positive tumor cells were regarded as having high indices.

### Definition of breast tumor subtypes

Breast cancer subtypes were defined according to Voduc and colleagues [34] with minor modifications, and were categorized as follows: luminal A (ER-positive or PR-positive, HER2-negative, Ki-67 < 14%), luminal B (ER-positive or PR-positive, HER2-negative, Ki-67 ≥ 14%; or ER-positive or PR-positive, HER2-positive), HER2-positive (ER-negative, PR-negative, HER2-positive), basal-like (ER-negative, PR-negative, HER2-negative, cytokeratin 5/6-positive and/or EGFR-positive) and triple-negative, nonbasal (negative for all markers). HER2 positivity was determined from the FISH results.

### Statistical analysis

After omitting all cases in which FISH analysis failed for all the genes (11 cases of invasive carcinoma, 13 cases of DCIS associated with invasive carcinoma and four cases of pure DCIS), a total of 427 invasive carcinomas, 203 DCIS associated with invasive carcinomas and 175 pure DCIS were informative for at least one gene. FISH failures were due to loss of tissue on the TMA, lack of tumor cells in the arrayed tissue or inadequate hybridization. Statistical significance was analyzed using Statistical Package SPSS version 15.0 for Windows (SPSS Inc., Chicago, IL, USA).

The chi-square test or Fisher's exact test was used when comparing gene amplification frequencies between groups and analyzing associations of gene amplification with clinicopathologic characteristics of tumors. The Spearman correlation test was used to analyze the association of co-amplification. For the 203 invasive carcinomas with DCIS components, McNemar tests were used to see whether the (paired) differences between gene amplification in the invasive and DCIS components of the same tumors were significantly different. Survival curves were estimated using the Kaplan-Meier product-limit method, and the significance of differences between survival curves was determined using the log-rank test. Covariates that were statistically significant in the univariate analysis were then included in the multivariate analysis using a Cox proportional hazards regression model; the hazard ratio (HR) and its 95% confidence interval (CI) were assessed for each factor. *P *< 0.05 was considered statistically significant. All *P *values reported were two-sided.

## Results

### Gene amplification in pure DCIS, DCIS associated with invasive carcinomas, and invasive carcinomas

**Figure 1:**
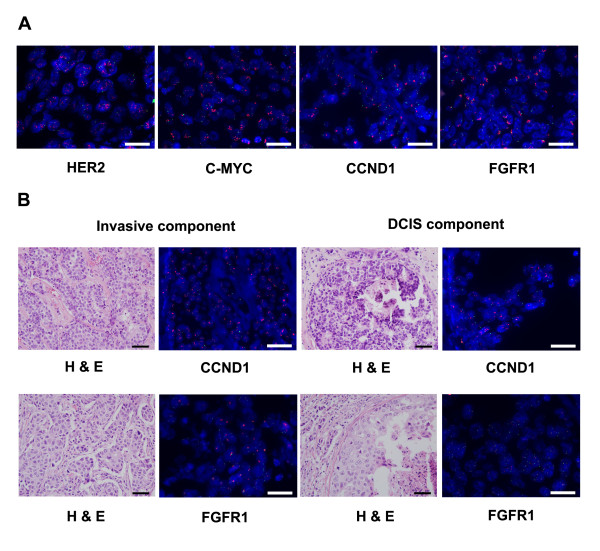
**Analysis of *HER2*, *C-MYC*, *CCND1 *and *FGFR1 *amplification by fluorescence *in situ *hybridization**. (**A**) Representative examples of *HER2*, *C-MYC*, *CCND1 *and *FGFR1 *amplification in pure ductal carcinoma *in situ *(DCIS). (**B**) Comparison of gene amplification status in the invasive and DCIS component of the same tumors. *CCND1 *is amplified in both the invasive and DCIS components of a tumor. However, *FGFR1 *is amplified in the invasive component but not in the DCIS component of a tumor. *HER2*, *C-MYC*, *CCND1 *and *FGFR1*-specific probes are red, and centromeric probes (chromosome 17 for *HER2*, chromosome 8 for *C-MYC *and *FGFR1*, and chromosome 11 for *CCND1*) are green. Scale bar: 25 μm. Magnification: ×400 (H & E) and ×1,000 (fluorescence *in situ *hybridization).

**Figure 2:**
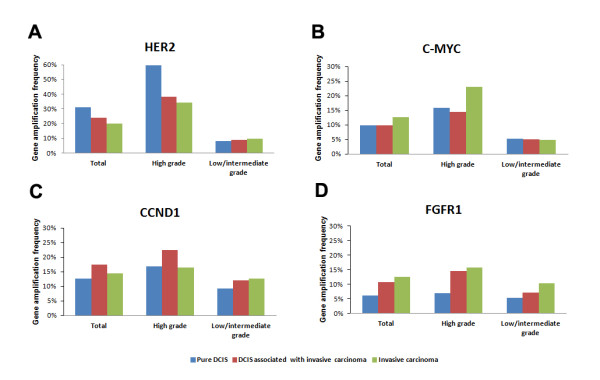
**Frequencies of *HER2*, *C-MYC*, *CCND1 *and *FGFR1 *amplification**. Frequencies of *HER2*, *C-MYC*, *CCND1 *and *FGFR1 *amplification in pure ductal carcinoma *in situ *(DCIS), DCIS associated with invasive carcinoma, and invasive carcinoma. (**A**) *HER2 *gene amplification is more frequent in pure DCIS than in invasive carcinomas in both total tumors and in high-grade tumors. **(B), (C) ***C-MYC *and *CCND1 *amplification rates do not differ significantly between groups. **(D) **The amplification rate of *FGFR1 *is higher in invasive carcinomas than in pure DCIS in both total tumors and high-grade tumors.

We measured the *HER2*, *C-MYC*, *CCND1 *and *FGFR1 *amplification status in pure DCIS, DCIS associated with invasive carcinomas, and invasive carcinomas (Figure 1). The frequency of *FGFR1 *amplification was higher in invasive carcinomas than in pure DCIS (12.5% vs. 6.0%, *P *= 0.020) (Table 2 and Figure 2). On the contrary, *HER2 *gene amplification was more frequent in pure DCIS than in invasive carcinomas (30.9% vs. 19.9%, *P *= 0.004). *C-MYC *and *CCND1 *amplification did not differ between the two groups. Similarly there was no significant difference in the amplification frequencies of *HER2*, *C-MYC*, *CCND1 *and *FGFR1 *in pure DCIS and DCIS associated with invasive carcinomas (Table 2 and Figure 2).

**Table 2:** Gene amplification frequencies in invasive carcinoma, DCIS associated with invasive carcinoma, and pure DCIS

Group	Gene	Pure DCIS	DCIS associated with invasive carcinoma	Invasive carcinoma	*P *value^a^	*P *value^b^
Total	*HER2*	54/175 (30.9)	48/202 (23.8)	85/427 (19.9)	0.004	0.122
	*C-MYC*	17/173 (9.8)	20/203 (9.9)	54/427 (12.6)	0.333	0.993
	*CCND1*	22/175 (12.6)	35/201 (17.4)	61/424 (14.4)	0.559	0.192
	*FGFR1*	10/168 (6.0)	21/196 (10.7)	52/417 (12.5)	0.020	0.105
High grade	*HER2*	46/77 (59.7)	39/102 (38.2)	63/183 (34.4)	< 0.001	0.004
	*C-MYC*	12/76 (15.8)	15/103 (14.6)	42/183 (23.0)	0.196	0.821
	*CCND1*	13/77 (16.9)	23/102 (22.5)	30/182 (16.5)	0.937	0.349
	*FGFR1*	5/74 (6.8)	14/97 (14.4)	28/179 (15.6)	0.056	0.114
Low/intermediate grade	*HER2*	8/98 (8.2)	9/100 (9.0)	22/230 (9.6)	0.687	0.834
	*C-MYC*	5/97 (5.2)	5/100 (5.0)	11/230 (4.8)	1.000	1.000
	*CCND1*	9/98 (9.2)	12/99 (12.1)	29/228 (12.7)	0.362	0.504
	*FGFR1*	5/94 (5.3)	7/99 (7.1)	23/225 (10.2)	0.158	0.614

Data presented as *n *(%). *P *values calculated using the chi-square test or Fisher's exact test. DCIS, ductal carcinoma *in situ*. ^a^Pure DCIS versus invasive carcinoma; ^b^Pure DCIS versus DCIS associated with invasive carcinoma.

In the next step, we compared gene amplification frequencies in DCIS and invasive carcinomas according to the grade of carcinoma (Table 2 and Figure 2), because DCIS are thought to be precursors of invasive cancers of similar grade. We found that *HER2 *and *C-MYC *amplification frequencies differed depending on the grade of carcinoma. In high-grade tumors, *HER2 *gene amplification frequency was also significantly higher in pure DICS than in invasive carcinoma (59.7% vs. 34.4%, *P *< 0.001). Conversely, the *FGFR1 *amplification frequency tended to be higher in invasive carcinoma than in pure DCIS (15.6% vs. 6.8%, *P *= 0.056). *HER2 *amplification was more frequent in high-grade pure DCIS than in DCIS associated with invasive carcinoma (59.7% vs. 38.2%, *P *= 0.004). For low/intermediate-grade tumors, however, there were no significant differences between the frequencies of gene amplification in pure DCIS, DCIS associated with invasive carcinomas, and invasive carcinomas.

We also evaluated co-amplification of genes and their associations in pure DCIS and invasive carcinoma (see Table S1 in Additional file 1). *CCND1 *amplification was correlated with *HER2 *and *FGFR1 *amplification in invasive carcinoma, but not in the pure DCIS.

### Comparison of gene amplification in the invasive and DCIS components of the same tumors

**Figure 3:**
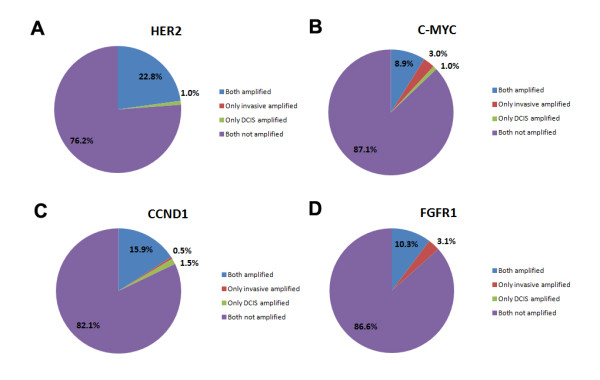
**Gene amplification status in invasive and ductal carcinoma *in situ *components of the same tumors**. **(A) ***HER2*, **(B) ***C-MYC*, **(C) ***CCND1 *and**(D) ***FGFR1 *amplification status in the invasive and ductal carcinoma *in situ *(DCIS) components agree in most cases. Discordant cases are found in 1.0% for *HER2*, 4.0% for *C-MYC*, 2.0% for *CCND1 *and 3.1% for *FGFR1 *in combined tissue microarray and whole-section examination.

To evaluate the role of gene amplification in the progression of DCIS to invasive carcinomas, we compared the gene amplification status of matched invasive and DCIS components in the 203 cases of invasive carcinomas with a DCIS component (Figure 3). The *HER2*, *C-MYC*, *CCND1 *and *FGFR1 *amplification status in the invasive and DCIS components agreed in most cases in the initial TMA examination. For the discrepant cases, we reanalyzed the gene amplification status of both components using all possible tumor sections with a DCIS component to find the potential area of gene amplification, and found that two of the discrepant cases in which only the invasive component showed gene amplification in initial TMA had a minor DCIS component with gene amplification in the whole section (Table S2 in Additional file 2).

The combined data for initial TMA and the whole-section examination revealed the concordance rate of 99.0% for HER2, 96.0% for *C-MYC*, 98.0% for *CCCND1 *and 96.9% for *FGFR1 *amplification (Figure 3). *HER2 *was amplified in both the invasive and DCIS components in 46 cases (22.8%), and in two cases (1.0%) there was amplification only in the DCIS component. *C-MYC *was amplified in 18 cases (8.9%) in both components, of which one case showed heterogeneous amplification in the DCIS component; that is, amplification was seen in some DCIS ducts but not in others. *C-MYC *amplification was present only in the invasive component in six cases (3.0%), in four of which amplification was variable. In two cases (1.0%), only the DCIS component showed heterogeneous *C-MYC *amplification. *CCND1 *was amplified in both components in 32 (15.9%) cases, in four of which amplification in the DCIS component was heterogeneous. *CCND1 *was amplified in only the DCIS component in three cases (1.5%) and in only the invasive component in one case (0.5%). Interestingly, amplification was heterogeneous in all four discordant cases. *FGFR1 *amplification was observed in both components in 20 cases (10.3%), in three of which amplification in the DCIS component was variable. In six cases (3.1%) only the invasive component showed *FGFR1 *amplification. *FGFR1 *amplification was thus found to be more frequent in the invasive component than in the DCIS component (*P *= 0.031).

### Association of gene amplification with the clinicopathologic characteristics of tumor

We also explored the associations between gene amplification and the clinicopathologic variables of the tumors (Table S3 in Additional file 3). In the invasive carcinomas, *HER2 *and *C-MYC *amplification were associated with the aggressive features of tumor, such as high histologic grade, ER/PR negativity, p53 overexpression and high Ki-67 proliferation index. *CCND1 *amplification was only correlated with ER positivity, and *FGFR1 *amplification was not associated with any clinicopatholgic features of the tumors. However, the association of *FGFR1 *amplification with high histologic grade was close to significance (*P *= 0.095). In pure DCIS, *HER2 *and *C-MYC *amplification were also associated with high nuclear grade and high Ki-67 proliferation index. However, no clinicopathologic variables of DCIS were associated with *FGFR1 *amplification.

Gene amplification frequencies were also associated with the tumor subtype (Table 3). Specifically, *CCND1 *amplification was not found in any of the progression stages of basal-like breast cancer. In invasive carcinoma, *C-MYC *amplification was most common in basal-like subtype and was more frequent in the luminal B subtype than in the luminal A subtype. *CCND1 *and *FGFR1 *amplification was most common in the luminal B subtype, being significantly higher than in the luminal A, HER2-positive and triple-negative, nonbasal subtypes. In pure DCIS, *CCND1 *and *FGFR1 *amplification was also most common in the luminal B subtype and tended to be more frequent in the luminal B subtype than in the luminal A subtype (*P *= 0.099 and *P *= 0.071, respectively). However, there were no significant differences in gene amplification frequencies between pure DCIS and invasive carcinoma within the individual subtypes, although *FGFR1 *amplification frequencies were higher (although not significantly so) in the invasive carcinomas than in the pure DCIS in all subtypes except the triple-negative, nonbasal subtype.

**Table 3:** Relationships between gene amplification and molecular subtypes

		Subtype					
		
Histologic stage	Gene	Luminal A	Luminal B	HER2-positive	Basal-like	TNNB	*P *value^a^
Invasive carcinoma	*C-MYC*	13/208 (6.3)*,**	15/103 (14.6)**	7/42 (16.7)	14/55 (25.5)*	5/19 (26.3)	< 0.001
	*CCND1*	19/205 (9.3)^†^	37/103 (35.9)^†,‡,§^	4/42 (9.5)^‡^	0/55 (0)^†^	1/18 (5.3)^§^	< 0.001
	*FGFR1*	21/202 (10.4)*^¶^*	21/100 (21.0)^¶,††,‡‡^	3/42 (7.1)^††^	7/55 (12.7)	0/18 (0)^‡‡^	0.025
Pure DCIS	*C-MYC*	6/98 (6.1)	4/24 (16.7)	6/34 (17.6)	1/9 (11.1)	0/8 (0)	0.198
	*CCND1*	11/99 (11.1)	6/24 (25.0)	4/35 (11.4)	0/9 (0)	1/8 (12.5)	0.298
	*FGFR1*	5/95 (5.3)	4/23 (17.4)	1/33 (3.0)	0/9 (0)	0/8 (0)	0.134

Data presented as *n *(%). *P *values calculated using the chi-square test or Fisher's exact test. DCIS, ductal carcinoma *in situ*; TNNB, triple negative, nonbasal. ^a^Between molecular subtypes. *P < 0.001, basal-like vs. luminal A. **P = 0.016, luminal B vs. luminal A for *C-MYC*. ^†^*P *< 0.001, luminal B vs. luminal A, luminal B vs. basal-like. ^‡^*P *= 0.001, luminal B vs. *HER2*-positive. ^§^*P *= 0.008, luminal B vs. TNNB for *CCND1*. *^¶^P = *0.012, luminal B vs. luminal A. ^††^*P *= 0.044, luminal B vs. *HER2*-positive. ^‡‡^*P *= 0.040, luminal B vs. TNNB for *FGFR1*.

### *FGFR1 *amplification as an independent prognostic factor for breast cancer progression

**Figure 4:**
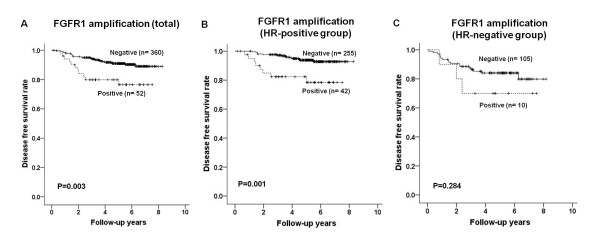
**Disease-free survival according to *FGFR1 *amplification status**. **(A) **Patients with *FGFR1 *amplified invasive breast cancer have significantly poorer disease-free survival than other patients. This finding applies to the hormone receptor (HR)-positive group **(B) **but not to the HR-negative group **(C)**.

We also investigated the prognostic role of gene amplification in invasive breast cancer. Most patients were treated by the standard practice guidelines and have been followed regularly after surgery. Among the 85 patients with *HER2*-amplified breast cancer, 20 patients (24%) received adjuvant trastuzumab therapy. There were five patients with initial metastases at the time of operation, and 422 patients were analyzed for disease-free survival. The median follow-up time was 5 years (range 1 to 8 years). There were 10 (2.4%) loco-regional recurrences and 34 (8.1%) distant metastases as first events. In Kaplan-Meier survival analyses, the patients with *FGFR1*-amplified breast cancer had shorter disease-free survival times than those without it (*P *= 0.003; Figure 4A). On the contrary, there were no survival differences associated with *HER-2*, *C-MYC *or *CCND1 *amplification (*P *= 0.160, *P *= 0.268, and *P *= 0.670, respectively). Subgroup analyses also revealed a difference in survival between the patients with and without *FGFR1*-amplified breast cancer in the hormone receptor-positive group (*P *= 0.001; Figure 4B), but not in the hormone receptor-negative group (*P *= 0.284; Figure 4C). Similarly, there was no survival differences with regard to *FGFR1 *amplification in the HER2-positive subtype (*P *= 0.514) and the basal-like subtype (*P *= 0.505).

In addition to *FGFR1 *amplification, high T stage (T1 to T2 vs. T3 to T4, *P *< 0.001), nodal metastasis (N0 vs. N1 to N3, *P *= 0.002), hormone receptor negativity (*P *= 0.004), p53 overexpression (*P *= 0.005) and high Ki-67 proliferation index (*P *= 0.006) were significantly associated with poor disease-free survival in all patients with invasive carcinoma. In multivariate analysis, only the T stage (pT1 to pT2 vs. pT3 to pT4, HR = 2.916, 95% CI = 1.254 to 6.781, *P *= 0.013), N stage (N0 vs. N1 to N3, HR = 2.585, 95% CI = 1.324 to 5.049, *P *= 0.005) and *FGFR1 *amplification (HR = 2.794, 95% CI = 1.375 to 5.678, *P *= 0.001) remained independent prognostic factors for disease-free survival. In the hormone receptor-positive group, the N stage (N0 vs. N1 to N3, HR = 4.514, 95% CI = 1.506 to 13.531, *P *= 0.007), p53 overexpression (HR = 2.757, 95% CI = 1.029 to 7.388, *P *= 0.044) and *FGFR1 *amplification (HR = 2.659, 95% CI = 1.042 to 6.785, *P *= 0.041) were identified as independent prognostic factors.

## Discussion

In this study we have shown that *FGFR1 *amplification is more frequent in invasive carcinoma than in pure DCIS, and in the invasive components of tumors with invasive and DICS components. In addition, *FGFR1 *amplification was found to be associated with decreased disease-free survival, suggesting a role for *FGFR1 *amplification in the progression of breast cancer including the *in situ *to invasive transition.

*FGFR1 *was suggested to be the target oncogene implicated in 8p11-12 amplification [15,35,36]. However, Gelsi-Boyer and colleagues reported that the 8p11-12 amplicon is much more complex, and is composed of at least four amplicons including 14 candidate oncogenes that can be amplified independently [37]. Not only *FGFR1 *but also other oncogenes in the 8p11-12 amplicon, such as *LSM1*, *BAG4 *and *C8orf4*, are therefore now accepted as contributing to oncogenesis [38,39]. Although *FGFR1 *is not widely accepted as the driver breast cancer oncogene affected in the 8p11-12 amplification, its amplification has been reported to be associated with poor prognosis, especially in patients with ER-positive tumors [14]. In this study, we confirmed the prognostic impact of *FGFR1 *amplification, especially in the hormone receptor-positive group. Furthermore, we showed that *FGFR1 *amplification is most frequently found in the luminal B subtype among the various breast cancer subtypes. No correlations were found between *FGFR1 *amplification and clinicopathologic features of the tumors. In the hormone receptor-positive group, however, *FGFR1 *amplification was associated with high histologic grade (*P *= 0.018), PR negativity (*P *= 0.017) and high Ki-67 proliferation index (*P *= 0.020) (data not shown). Recently, in accord with our findings, Turner and colleagues reported that FGFR1 signaling suppressed PR expression and that *FGFR1*-amplified cancers had higher Ki-67 proliferation indices and were most often found in the luminal B subtype, accounting for 16 to 27% of their series [17]. They also demonstrated that *FGFR1 *amplification increased resistance to endocrine therapy. Taken together, these findings suggest that *FGFR1 *amplification makes an important contribution to the aggressive phenotype of hormone receptor-positive breast cancer.

Although *FGFR1 *amplification has been shown to be associated with breast cancer progression, there have been no studies of its association with the *in situ *to invasive transition. In our study, *FGFR1 *amplification was more frequent in invasive carcinoma than in pure DCIS, and tended to be higher in invasive carcinomas than in pure DCIS in the high-grade tumors. More importantly, *FGFR1 *amplification was more frequent in the invasive components of tumors than in the corresponding DCIS components. The mechanism by which *FGFR1 *amplification induces invasion of DCIS is unknown. However, in the mouse mammary tumor virus-inducible *FGFR1 *transgenic mouse model, sustained activation of *FGFR1 *in the mouse mammary epithelium induces alveolar hyperplasia and invasive lesions, which are associated with extracellular matrix remodeling and vascular branching in the stroma adjacent to these lesions [40]. The same authors have developed an *in vitro *three-dimensional HC11 mouse mammary epithelial cell culture model expressing a drug-inducible *FGFR1 *and have demonstrated that inducible *FGFR1 *activation results in a gain of invasive properties and promotes the epithelial-mesenchymal transition, which is caused by induction of matrix metalloproteinase-3 [41]. *FGFR1 *amplification, and hence increased FGFR1 signaling, therefore seems to contribute to early breast cancer invasion; that is, the *in situ *to invasive transition. This result may have clinical implications, because DCIS with *FGFR1 *amplification is more likely to progress to invasive carcinoma. In this study, we focused on the role of genetic alterations in the progression of DCIS to invasive carcinoma. However, a growing body of evidence suggests that the transition of DCIS to invasive carcinoma is strongly dependent on the tumor microenvironment, particularly on the myoepithelial cells and cancer-associated fibroblasts [42,43]. Further studies will therefore be needed to elucidate the effect of *FGFR1 *amplification in epithelial cells on the tumor microenvironment and epithelial-stromal interactions.

We found no differences in the amplification frequencies of *C-MYC *and *CCND1 *between DCIS, DCIS associated with invasive carcinoma, and invasive carcinoma. Burkhardt and colleagues recently examined the amplification frequencies of *HER2*, *ESR1*, *CCND1 *and *MYC *in a large series of pure DCIS and DCIS associated with invasive carcinomas, and also found no significant differences between them [28]. However, we obtained different results for *HER2 *gene amplification. This amplification was significantly more frequent in pure DCIS than in invasive carcinomas, especially in high-grade tumors, and other workers have obtained the same result [44,45]. Amplification of *HER2*, *C-MYC *and *CCND1 *therefore seems to play a role in the early development of breast cancer, but not in the progression of DCIS to invasive carcinomas.

In a comparison of the matched DCIS and invasive components of the same tumors we demonstrated that the genetic changes in the two components were similar in terms of *HER2*, *C-MYC *and *CCND1 *gene amplification. However, we detected amplification in the DCIS components but not in the invasive carcinoma components in a few cases for *HER2 *(*n *= 2), *C-MYC *(*n *= 2) and *CCND1 *(*n *= 3). Interestingly, the two cases with *C-MYC *amplification and the three cases with *CCND1 *amplification in only the DCIS component showed heterogeneous amplification. Four of the 32 cases with *CCND1 *amplification in both components and three of the 20 cases that had *FGFR1 *amplification in both components also had heterogeneous amplification in the DCIS component. Furthermore, in pure DCIS, heterogeneity of gene amplification was found in 3.7% (2/52) for *HER2*, 11.8% (2/17) for *C-MYC*, 22.7% (5/22) for *CCND1 *and 10% (1/10) for *FGFR1 *amplified cases (data not shown). These findings suggest that intra-tumoral genetic heterogeneity is already present in the DCIS and that progression of DCIS to invasive carcinomas may result from selection of subpopulations of tumor cells. In a previous study we reported that the differences in molecular subtypes among the invasive tumor foci of multifocal/multicentric breast cancers were associated with mixed molecular subtypes in the DCIS components, suggesting that heterogeneity within the DCIS followed by selection of different clones might give rise to the different phenotypes in multicentric/multifocal breast cancers [46]. Supporting this concept, Hernandez and colleagues recently performed comparative analyses of known cancer genes using microarray-based comparative genomic hybridization and Sequenom MassARRAY (OncoCarta Panel v 1.0, Sequenom, San Diego, CA, USA) in matched DCIS and adjacent invasive carcinomas, and suggested that although the modal populations of both components were similar at the genetic level, the progression from DCIS to invasive ductal carcinoma was driven in some cases by selection of nonmodal clones with specific genetic aberrations [47].

The different breast cancer subtypes have been suggested to have distinct patterns of copy number alterations. While higher numbers of gains/losses have been associated with the basal-like subtype, high-level DNA amplification has been found in the luminal B and HER2-positive subtypes [48], and candidate oncogenes have been identified in chromosomal regions 1q21-23, 10p14 and 12p13 for basal-like breast cancers, but in regions 1q21-23, 8p12-q21, 11q13 and 16p12-13 for luminal breast cancers [4]. Moreover, *CCND1 *amplification on 11q13 has been reported to be associated with luminal subtypes [16], and in agreement with this we found that *CCND1 *and *FGFR1 *amplification was most frequent in the luminal B subtype whereas *CCND1 *amplification was absent from all of the basal-like breast cancers.

## Conclusion

In summary, we have studied the amplification frequencies of *HER2*, *C-MYC*, *CCND1 *and *FGFR1 *in a large series of pure DCIS, DCIS associated with invasive carcinoma, and invasive carcinomas, to investigate the role of gene amplification in the progression of DCIS to invasive carcinoma. The amplification frequencies of *C-MYC *and *CCND1 *did not differ between pure DCIS and invasive carcinomas, and *HER2 *amplification was more frequent in pure DCIS. The *HER2*, *C-MYC*, *CCND1 and FGFR1 *amplification status was in most cases concordant in the matched invasive and DCIS components of the same tumors, pointing to early roles in the development of breast cancer. However, *FGFR1 *amplification was more frequent in invasive carcinomas than in pure DCIS, and in the invasive components of the same tumors. Furthermore, *FGFR1 *amplification was found to be an independent prognostic factor for disease-free survival. Our results therefore suggest that *FGFR1 *amplification play an important role in the progression of breast cancer, including the *in situ *to invasive transition, as well as initiation.

## Abbreviations

CEP: chromosome enumeration probe; CI: confidence interval; DCIS: ductal carcinoma *in situ*; EGFR: epidermal growth factor receptor; ER: estrogen receptor; FGFR1: fibroblast growth factor receptor 1; FISH: fluorescence *in situ *hybridization; H & E: hematoxylin and eosin; HER2: human epidermal growth factor receptor 2; HR: hazard ratio; LSI: locus specific identifier; PR: progesterone receptor; TMA: tissue microarray.

## Competing interests

The authors declare that they have no competing interests.

## Authors' contributions

MHJ participated in the interpretation and analysis of data and drafted the manuscript. EJK carried out the experiments and participated in the interpretation of the data. YC and HEL participated in the acquisition and interpretation of pathologic data. YJK, JHK, EK, S-WK and IAK participated in the acquisition of clinical data. SYP conceived of the study, and participated in its design and was responsible for preparation of the manuscript. All authors read and approved the final manuscript.

## Supplementary Material

Additional file 1 **Table S1 presenting frequencies of co-amplification of genes (A) and their correlations (B) in invasive carcinoma and pure DCIS**. Click here for file

Additional file 2 **Table S2 presenting a comparison of initial TMA and whole-section examination for gene amplification status in discrepant cases for invasive and *in situ *components of a same tumor**. Click here for file

Additional file 3 **Table S3 presenting correlation of *HER2*, *C-MYC*, *CCND1 *and *FGFR1 *amplification with clinicopathologic characteristics in invasive (A) and *in situ *(B) breast carcinomas**. Click here for file
