# Supplementary material for: FGFR1 is amplified during the progression of in situ to invasive breast carcinoma
Source: Breast Cancer Res. 2012 Aug 3;14(4):R115. doi: 10.1186/bcr3239 (PMC3680930; doi:10.1186/bcr3239)
Supplement: Additional file 1 — Table S1 presenting frequencies of co-amplification of genes (A) and their correlations (B) in invasive carcinoma and pure DCIS. [file bcr3239-S1.DOC]

**Table S1. Frequencies of co-amplification of genes (A) and their correlations (B) in invasive carcinoma and pure DCIS**

A

| **Histologic stage** | **Gene** | **Frequency of co-amplification (%)** |
| --- | --- | --- |
| **Invasive carcinoma** | HER2 & C-MYC | 3.5 |
|  | HER2 & CCND1 | 5.0 |
|  | HER2 & FGFR1 | 3.4 |
|  | C-MYC & CCND1 | 2.8 |
|  | C-MYC & FGFR1 | 2.6 |
|  | CCND1 & FGFR1 | 3.4 |
| **Pure DCIS** | HER2 & C-MYC | 4.6 |
|  | HER2 & CCND1 | 4.0 |
|  | HER2 & FGFR1 | 2.4 |
|  | C-MYC & CCND1 | 0.6 |
|  | C-MYC & FGFR1 | 1.2 |
|  | CCND1 & FGFR1 | 1.8 |

B

| **Histologic stage** | **Correlation coefficient*** | **HER2** | **C-MYC** | **CCND1** | **FGFR1** |
| --- | --- | --- | --- | --- | --- |
| **Invasive carcinoma** | **HER2** | 1.000 |  |  |  |
|  | **C-MYC** | 0.075 (0.122) | 1.000 |  |  |
|  | **CCND1** | 0.147 (0.002) | 0.085 (0.079) | 1.000 |  |
|  | **FGFR1** | 0.064 (0.194) | 0.092 (0.060) | 0.138 (0.005) | 1.000 |
| **Pure DCIS** | **HER2** | 1.000 |  |  |  |
|  | **C-MYC** | 0.118 (0.123) | 1.000 |  |  |
|  | **CCND1** | 0.008 (0.917) | -0.068 (0.376) | 1.000 |  |
|  | **FGFR1** | 0.053 (0.497) | 0.081 (0.297) | 0.133 (0.085) | 1.000 |

*Spearman correlation coefficient; Numbers in parentheses indicate p-value.
