# Supplementary material for: FGFR1 is amplified during the progression of in situ to invasive breast carcinoma
Source: Breast Cancer Res. 2012 Aug 3;14(4):R115. doi: 10.1186/bcr3239 (PMC3680930; doi:10.1186/bcr3239)
Supplement: Additional file 2 — Table S2 presenting a comparison of initial TMA and whole-section examination for gene amplification status in discrepant cases for invasive and in situ components of a same tumor. [file bcr3239-S2.DOC]

**Table S2.** **Comparison of initial TMA and whole section examination for gene amplification status in discrepant cases for invasive and in situ components of a same tumor**

| **Gene** | **Case No** | **TMA** | | **Whole section** | |
| --- | --- | --- | --- | --- | --- |
|  |  | **Invasive component** | **DCIS component** | **Invasive component** | **DCIS component** |
| **HER2** | 21 | - | + | - | + |
|  | 389 | - | + | - | + |
| **C-MYC** | 112 | +/- | - | +/- | - |
|  | 122 | +/- | - | +/- | - |
|  | 150 | +/- | - | +/- | - |
|  | 188 | + | - | + | +/- |
|  | 209 | +/- | - | +/- | - |
|  | 353 | + | - | + | NA |
|  | 373 | + | - | + | - |
|  | 364 | - | + | - | +/- |
|  | 378 | - | + | - | +/- |
| **CCND1** | 265 | +/- | - | +/- | - |
|  | 117 | - | +/- | - | +/- |
|  | 377 | - | +/- | - | - |
|  | 405 | - | +/- | - | +/- |
| **FGFR1** | 21 | + | - | + | - |
|  | 56 | + | - | + | - |
|  | 65 | + | - | + | - |
|  | 209 | + | - | + | - |
|  | 382 | + | - | +/- | - |
|  | 399 | + | - | + | - |
|  | 408 | + | - | + | +/- |

+, amplification; -, no amplification; +/-, heterogeneous amplification; NA, not available
