# Supplementary material for: FGFR1 is amplified during the progression of in situ to invasive breast carcinoma
Source: Breast Cancer Res. 2012 Aug 3;14(4):R115. doi: 10.1186/bcr3239 (PMC3680930; doi:10.1186/bcr3239)
Supplement: Additional file 3 — Table S3 presenting correlation of HER2, C-MYC, CCND1 and FGFR1 amplification with clinicopathologic characteristics in invasive (A) and in situ (B) breast carcinomas. [file bcr3239-S3.DOC]

**Table S3. Correlation of *HER2, C-MYC, CCND1* and *FGFR1* amplification with clinicopathologic characteristics in invasive (A) and in situ (B) breast carcinomas.**

**A**

| **Clinicopathologic**  **Characteristics** | | **HER2 amplification** | | **p-value** | **C-MYC amplification** | | **p-value** | **CCND1 amplification** | | **p-value** | **FGFR1 amplification** | | **p-value** |
| --- | --- | --- | --- | --- | --- | --- | --- | --- | --- | --- | --- | --- | --- |
|  | | **Negative**  **(n = 342)** | **Positive**  **(n = 85)** |  | **Negative**  **(n = 373)** | **Positive (n = 54)** |  | **Negative**  **(n = 363)** | **Positive**  **(n = 61)** |  | **Negative**  **(n = 365)** | **Positive**  **(n = 52)** |  |
| **Age (yr)** | | 50.0 ± 11.7 | 50.9 ± 10.6 | 0.492 | 50.3 ± 11.7 | 49.0 ± 10.2 | 0.440 | 50.1 ± 11.7 | 51.4 ± 10.4 | 0.414 | 50.1 ± 11.1 | 50.3 ± 14.0 | 0.929 |
| **T stage** | **T1-T2** | 326 (95.3) | 80 (94.1) | 0.584 | 356 (95.4) | 50 (92.6) | 0.322 | 346 (95.3) | 57 (93.4) | 0.523 | 347 (95.1) | 49 (94.2) | 0.736 |
|  | **T3-T4** | 16 (4.7) | 5 (5.9) |  | 17 (4.6) | 4 (7.4) |  | 17 (4.7) | 4 (6.6) |  | 18 (4.9) | 3 (5.8) |  |
| **N stage** | **N0** | 179 (52.3) | 45 (52.9) | 0.921 | 193 (51.7) | 31 (57.4) | 0.436 | 190 (52.3) | 32 (52.5) | 0.986 | 192 (52.6) | 26 (50.0) | 0.725 |
|  | **N1-N3** | 163 (47.7) | 40 (47.1) |  | 180 (48.3) | 23 (42.6) |  | 173 (47.7) | 29 (47.5) |  | 173 (47.4) | 26 (50.0) |  |
| **Histologic** | **I & II** | 209 (63.7) | 22 (25.9) | <0.001 | 220 (61.1) | 11 (20.8) | <0.001 | 200 (57.0) | 29 (49.2) | 0.263 | 203 (57.5) | 23 (45.1) | 0.095 |
| **grade** | **III** | 119 (36.3) | 63 (74.1) |  | 140 (38.9) | 42 (79.2) |  | 151 (43.0) | 30 (50.8) |  | 150 (42.5) | 28 (54.9) |  |
| **Angiolymphatic** | **Absent** | 119 (58.2 ) | 45 (52.9) | 0.382 | 214 (57.4) | 30 (55.6) | 0.801 | 213 (58.7) | 29 (47.5) | 0.104 | 208 (57.0) | 27 (51.9) | 0.491 |
| **invasion** | **Present** | 143 (41.8) | 40 (47.1) |  | 159 (42.6) | 24 (44.4) |  | 150 (41.3) | 32 (52.5) |  | 157 (43.0) | 25 (48.1) |  |
| **ER** | **Negative** | 80 (23.4) | 45 (52.9) | <0.001 | 99 (26.5) | 26 (48.1) | 0.001 | 118 (32.5) | 7 (11.5) | 0.001 | 113 (31.0) | 11 (21.2) | 0.148 |
|  | **Positive** | 262 (76.6) | 40 (47.1) |  | 274 (73.5) | 28 (51.9) |  | 245 (67.5) | 54 (88.5) |  | 252 (69.0) | 41 (78.8) |  |
| **PR** | **Negative** | 118 (34.5) | 53 (62.4) | <0.001 | 138 (37.0) | 33 (61.1) | 0.001 | 151 (41.6) | 19 (31.1) | 0.123 | 145 (39.7) | 23 (44.2) | 0.536 |
|  | **Positive** | 224 (65.5) | 32 (37.6) |  | 235 (63.0) | 21 (38.9) |  | 212 (58.4) | 42 (68.9) |  | 220 (60.3) | 29 (55.8) |  |
| **Ki-67 index** | **<20%** | 230 (67.3) | 25 (29.4) | <0.001 | 236 (63.3) | 19 (35.2) | <0.001 | 220 (60.6) | 32 (52.5) | 0.231 | 221 (60.5) | 26 (50.0) | 0.148 |
|  | **≥20%** | 112 (32.7) | 60 (70.6) |  | 137 (36.7) | 35 (64.8) |  | 143 (39.4) | 29 (47.5) |  | 144 (39.5) | 26 (50.0) |  |
| **P53** | **Negative** | 227 (81.0) | 48 (56.5) | <0.001 | 291 (78.0) | 34 (63.0) | 0.015 | 276 (76.0) | 47 (77.0) | 0.863 | 280 (76.7) | 39 (75.0) | 0.785 |
|  | **Positive** | 65 (19.0) | 37 (43.5) |  | 82 (22.0) | 20 (37.0) |  | 87 (24.0) | 14 (23.0) |  | 85 (23.3) | 13 (25.0) |  |

**B**

| **Clinicopathologic**  **Characteristics** | | **HER2 amplification** | | **p-value** | **C-MYC amplification** | | **p-value** | **CCND1 amplification** | | **p-value** | **FGFR1 amplification** | | **p-value** |
| --- | --- | --- | --- | --- | --- | --- | --- | --- | --- | --- | --- | --- | --- |
|  | | **Negative**  **(n = 121)** | **Positive**  **(n = 54)** |  | **Negative**  **(n = 156)** | **Positive (n = 17)** |  | **Negative**  **(n = 153)** | **Positive**  **(n = 22)** |  | **Negative**  **(n = 158)** | **Positive**  **(n = 10)** |  |
| **Age (yr)** | | 49.5 ± 12.1 | 51.9 ± 10.5 | 0.213 | 50.9 ± 11.7 | 44.0 ± 9.1 | 0.020 | 50.7 ± 11.1 | 46.9 ± 15.0 | 0.151 | 50.2 ± 11.9 | 47.7 ± 7.4 | 0.515 |
| **Size (cm)** | | 3.2 ± 2.2 | 3.2 ± 1.9 | 0.931 | 3.1 ± 2.0 | 4.2 ± 2.4 | 0.044 | 3. 2 ± 2.2 | 3.5 ± 1.8 | 0.563 | 3.3 ± 2.1 | 2.4 ± 1.3 | 0.235 |
| **Nuclear grade** | **1 & 2** | 90 (74.4) | 8 (14.8) | <0.001 | 92 (59.0) | 5 (29.4) | 0.020 | 89 (58.2) | 9 (40.9) | 0.127 | 89 (56.3) | 5 (50.0) | 0.751 |
|  | **3** | 31 (25.6) | 46 (85.2) |  | 64 (41.0) | 12 (70.6) |  | 64 (41.8) | 13 (59.1) |  | 69 (43.7) | 5 (50.0) |  |
| **ER** | **Negative** | 19 (15.7) | 36 (66.7) | <0.001 | 47 (30.1) | 7 (41.2) | 0.351 | 49 (32.0) | 6 (27.3) | 0.653 | 52 (32.9) | 1 (10.0) | 0.173 |
|  | **Positive** | 102 (84.3) | 18 (33.3) |  | 109 (69.9) | 10 (58.8) |  | 104 (68.0) | 16 (72.7) |  | 106 (67.1) | 9 (90.0) |  |
| **PR** | **Negative** | 29 (24.0) | 44 (81.5) | <0.001 | 63 (40.4) | 9 (52.9) | 0.319 | 67 (43.8) | 6 (27.3) | 0.142 | 66 (41.8) | 4 (40.0) | 1.000 |
|  | **Positive** | 92 (76.0) | 10 (18.5) |  | 93 (59.6) | 8 (47.1) |  | 86 (56.2) | 16 (72.7) |  | 92 (58.2) | 6 (60.0) |  |
| **Ki-67 index** | **<20%** | 115 (95.0) | 36 (66.7) | <0.001 | 139 (89.1) | 11 (64.7) | 0.013 | 135 (88.2) | 16 (72.7) | 0.088 | 139 (88.0) | 7 (70.0) | 0.127 |
|  | **≥20%** | 6 (5.0) | 18 (33.3) |  | 17 (10.9) | 6 (35.3) |  | 18 (11.8) | 6 (27.3) |  | 19 (12.0) | 3 (30.0) |  |
| **P53** | **Negative** | 104 (86.0) | 34 (63.0) | 0.001 | 122 (78.2) | 14 (82.4) | 1.000 | 126 (82.4) | 12 (54.5) | 0.009 | 126 (79.7) | 7 (70.0) | 0.436 |
|  | **Positive** | 17 (14.0) | 20 (37.0) |  | 34 (21.8) | 3 (17.6) |  | 27 (17.6) | 10 (45.5) |  | 32 (20.3) | 3 (30.0) |  |

Numbers in parentheses indicate percentage. P value was calculated using Chi-square or Fisher’s exact test. * Histologic grade was available for 413 cases.
